# Epigenetics and immune cells in medulloblastoma

**DOI:** 10.3389/fgene.2023.1135404

**Published:** 2023-03-10

**Authors:** Francesca Gorini, Marco Miceli, Pasqualino de Antonellis, Stefano Amente, Massimo Zollo, Veronica Ferrucci

**Affiliations:** ^1^ Department of Molecular Medicine and Medical Biotechnology (DMMBM), University of Naples, Naples, Italy; ^2^ CEINGE Biotecnologie Avanzate “Franco Salvatore”, Naples, Italy; ^3^ DAI Medicina di Laboratorio e Trasfusionale, ‘AOU Federico II Policlinico, Naples, Italy

**Keywords:** medulloblastoma, epigenetics, brain tumor microenvironment, immune cells, immunotherapeutics

## Abstract

Medulloblastoma (MB) is a highly malignant childhood tumor of the cerebellum. Transcriptional and epigenetic signatures have classified MB into four molecular subgroups, further stratified into biologically different subtypes with distinct somatic copy-number aberrations, driver genes, epigenetic alterations, activated pathways, and clinical outcomes. The brain tumor microenvironment (BTME) is of importance to regulate a complex network of cells, including immune cells, involved in cancer progression in brain malignancies. MB was considered with a “cold” immunophenotype due to the low influx of immune cells across the blood brain barrier (BBB). Recently, this assumption has been reconsidered because of the identification of infiltrating immune cells showing immunosuppressive phenotypes in the BTME of MB tumors. Here, we are providing a comprehensive overview of the current status of epigenetics alterations occurring during cancer progression with a description of the genomic landscape of MB by focusing on immune cells within the BTME. We further describe how new immunotherapeutic approaches could influence concurring epigenetic mechanisms of the immunosuppressive cells in BTME. In conclusion, the modulation of these molecular genetic complexes in BTME during cancer progression might enhance the therapeutic benefit, thus firing new weapons to fight MB.

## Introduction

Medulloblastoma (MB) is a highly malignant tumor of the cerebellum classified as CNS WHO grade 4, (Rausch et al., 2012; Louis et al., 2016). MB comprises ∼73.3% of childhood intracranial embryonal cancers with a median peak incidence of 7.2 years of age, 1.8:1 male:female ratio, and no differences in incidence across ethnicities (Ezzat et al., 2016; Northcott et al., 2017; Northcott et al., 2019).

MB is not a single disease entity and different histopathological morphologies have been distinguished: classic, desmoplastic/nodular (DN), MB with extensive nodularity (MBEN), and large cell and anaplastic (LC/A). The last morphology comprises ∼10% of cases and is in general associated with poor outcomes (Ellison, 2010).

According to the current consensus, MB segregates into four biologically distinct molecular subgroups that are WNT, sonic hedgehog (SHH), Group 3 (GR3) and Group 4 (GR4) with distinctive transcriptional and chromosome aberrations with prognostic significance (Taylor et al., 2012). Among the subgroups, GR3 and GR4 MB (i.e., “non-WNT/non-SHH”) represent over two-thirds of all MB patients and show the worst prognosis to their higher tendency to metastasize, thus representing a complex challenge (Rausch et al., 2012; Louis et al., 2016).

Recently, using different -omics approaches, a molecular classification has been further refined. Through an unsupervised class discovery based on n.428 MB profiled by DNA methylation array, seven molecular subtypes of MB were established: one WNT subgroup, two age-dependent SHH (SHH-Infant <4.3 years, and SHH-Child ≥4.3 years), two GR3 (high-risk GR3 and low-risk GR3), and two GR4 (high-risk GR4 and low-risk GR4) subtypes (Schwalbe et al., 2017).

More recently, MB subgroups have been further stratified through an integrative spectral clustering with the “similarity network fusion” approach applied to genome-wide DNA methylation and gene expression data, taking into account also somatic copy-number alterations and clinical features across n.763 primary MB samples (Cavalli et al., 2017). Thus, the boundaries between the 4 MB subgroups have been more precisely refined, by identifying twelve subtypes with distinct somatic copy-number aberrations, differentially activated pathways, and disparate clinical outcomes: two WNT (−α, −β), four SHH (−α, −β, −γ, −δ), three GR3 (−α, −β, −γ), and three GR4 (−α, −β, −γ) MB subtypes (Cavalli et al., 2017).

Additionally, GR3 and GR4 MB subgroups have been further stratified into eight subtypes by considering cytogenetic and focal copy number variations through a high-resolution subclassification approaches applied to n.1501 MB specimens collected from three published cohorts (Cavalli et al., 2017) (Ezzat et al., 2016; Northcott et al., 2017; Schwalbe et al., 2017; Northcott et al., 2019) and independent n.153 tumors (Sharma et al., 2019).

Furthermore, among these “non-WNT/non-SHH” MB, “low-risk” and a “very high-risk” subtypes have been identified showing a five-year progression-free survival (PFS) of 94% ± 5.7% and 29% ± 6.1%, respectively, through the integration of the whole chromosomal aberration (WCA) phenotypes with MYC/N amplification, subgroup definition and clinical features (i.e., age and metastatic status) (Goschzik et al., 2018; Mynarek et al., 2022).

To date, the diagnosis of MB requires the combination of both histological feature and molecular subgroups. WNT MB are mostly found with a classic histology. SHH MB has been described with all the morphologies with different percentages: classic ∼43%, DN ∼33% (mostly associated with TP53 wild-type), MBEN ∼10% and LC/A in ∼17% of tumors (frequently associated with mutations in TP53). GR3 and GR4 MBs are mostly classic or with an LC/A morphology (Rausch et al., 2012; Louis et al., 2016).

However, despite the molecular phenotyping integrated with the histological variants, the “standard-of-care” therapy for MB is multimodal and generally consists of maximal surgical resection followed by radiation (e.g., cranio-spinal irradiation [CSI], for those “standard-low risk” patients >3 age) and adjuvant chemotherapy, specifically designed according to patient age and risk stratification (Ezzat et al., 2016; Northcott et al., 2017; Northcott et al., 2019). The five-years overall survival (OS) for the “standard-risk” patients (i.e., non-metastatic patients >3 years of age at diagnosis completely resected) is 70%–85%. In contrast, those patients classified as “high risk” (<3 years of age, with a subtotal resection and/or with metastasis at diagnosis) show a five-years OS <70% (Ezzat et al., 2016; Northcott et al., 2017; Northcott et al., 2019; Thompson et al., 2016). Furthermore, the prognosis is very poor (five-years survival <10%) when MB recurs. This has been already observed since 2013 (Zollo, 2013). Although the survival of MB is slowly improving, the therapeutic approaches currently used for MB management have a high toxicity rate (mostly due to radiation therapy), and the survivors are often left with devastating long-term side effects, including permanent neurocognitive disability, neuroendocrine dysfunction, growth disturbances, infertility, growth deformities and secondary malignancy (Ezzat et al., 2016; Northcott et al., 2017; Northcott et al., 2019). Thus, the integration of molecular and histopathological features aimed to reduce the radiation intensity are the main goal of ongoing studies and/or clinical trials (NCT01878617, (Khan et al., 2021); NCT02066220 (Thompson et al., 2020),NCT01878617, SJMB12; NCT02066220, PNET5; NCT02724579; ACNS1422). Notwithstanding the foregoing, further efforts to ameliorate MB management are strongly demanded.

Of much interest, integrated data from gene expression, DNA sequencing, and methylation studies, revealed that MB harbor a paucity of genetic alterations in oncogenes and tumor suppressors with mutations also occurring in epigenetic regulators, thus suggesting epigenetic modulation (Cavalli et al., 2017) (Ezzat et al., 2016; Northcott et al., 2017; Schwalbe et al., 2017; Northcott et al., 2019). Indeed, alterations in chromatin modifiers genes appear to be a common and converging mechanism underlying MB pathogenesis.

Recently, the brain tumor microenvironment (BTME), comprising microglia, immune cells, and the blood-brain barrier, has been recognized as a critical regulator of cancer progression in brain malignancies, including MB (Quail and Joyce, 2017). Thus, in addition to the aberrant epigenetic alterations in tumorigenic cells, epigenetic marks are also present in those immune cells (including lymphocytes and tumor-associated macrophages [TAMs]), which altogether contributes to generate an immunosuppressive environment that favors the tumor growth (Marks et al., 2016; Sylvestre et al., 2020).

Thus, a more comprehensive understanding of the genetic and epigenetic microenvironmental interconnection would be needed, to amply the range of targeted therapeutic strategies against MB.

Mostly this review underlies the importance of the immune response in the BTME of MB. Therefore, the main aim is to give an overview of the epigenetic alterations affecting immune cells in the BTME thus causing an immunosuppressive phenotype that contributes to cancer development. We here discuss further how immunotherapy combined with epigenetics treatment would open new possibilities for therapeutic intervention.

### The genetic landscape of MB subtypes and their cellular origins

Gene mutation studies, chromosomal abnormal modification, epigenetics (including DNA methylation), and transcriptomic studies have recently stratified MB into different genetic subtypes: WNT (α, β), SHH (α, β, γ, δ), GR3 (α, β, γ), and GR4 (α, β, γ) (Cavalli et al., 2017).

WNT MB subgroup accounts for ∼10% of all MB diagnoses and is infrequently metastatic at diagnosis. WNT-α is mainly comprised of children (median 10 years at diagnosis), has ubiquitous monosomy 6, and shows a similar survival as WNT-β that is mainly enriched for older patients (median 20 yours at diagnosis) who are mostly diploid for chromosome 6 (Cavalli et al., 2017). The prognosis is excellent in terms of 5 years OS in patients <16 years of age (Ellison et al., 2005).

SHH MB is the dominant subgroup in infants <3 years of age and represents only ∼10–15% of MB during childhood and adolescence (Kool et al., 2012). In fact, epigenetic studies and DNA methylation profiling have shown biologically distinct subtypes between infant and childhood stages (Schwalbe et al., 2017). The prognosis for SHH MB mostly relies on the subtype considering patient age, tumor histology, metastatic status, and genotype. SHHα tumor primarily occurs in children ranging from 3 to 16 years (median 8 years). Regarding their genetic and cytogenetic hallmarks, these tumors show amplification of MYCN, GLI2 and YAP1, loss of chromosomes 9q, 10q and 17p and are enriched of TP53 mutations that act as a prognostic indicator in SHH (Cavalli et al., 2017). SHH-β tumors mostly involve infants with a median of 1.9 years of age at diagnosis. This subtype is frequently metastatic carrying PTEN deletions, thus showing worse overall survival compared to the other SHH subtype. Conversely, SHH- γ also occurs in infants (median of 1.3 aged at diagnosis) but shows the absence of recurrent amplifications and gains. SHH-δ is more frequent in adults (median age is 26 years) and shows a more favorable prognosis as compared to the others SHH subtype. TERT promoter mutations are enriched in SHH-γ/-α MB (Cavalli et al., 2017).

GR3 MB is considered the most aggressive subgroup because of the high metastatic potential and the poor survival rate. Three GR3 MB subtypes have been identified (α, β, γ). GR3-α and GR3-γ have a similar tendency to metastasize. GR3-α is enriched for chromosome 8q (MYC locus at 8q24) loss and mostly involves infants <3 years of age at diagnosis compared to the other GR3 subtypes. GR3- β tumors are characterized by GFI1 and GFI1B oncogenes activation, which had been previously reported to act as driver genes of GR3 MB through enhancer hijacking process (*via* focal gains and losses on chromosomes 1 and 9; (Northcott et al., 2014). This subtype is also enriched for OTX2 amplification and DDX31 loss. GR3-γ subtype shows the worst prognosis, with a trend to the enrichment of i17q. Despite the high frequency of chromosome 8q gain responsible for and increased MYC copy number, GR3-γ has a poor prognosis independent of MYC amplification (Cavalli et al., 2017). Recently, a new metastatic axis (independent of c-MYC amplification) was dissected in MB GR3 driven by Prune1 gene, whose expression levels were high in metastatic MB subgroups (i.e., GR3, GR4 MB; (Ferrucci et al., 2018; Bibbo et al., 2021). In detail, Prune1, through its binding to NDPK-A, promotes the canonical TGF-β pathway with OTX2 and SNAIL upregulation, decreases PTEN levels, and enhances N-cadherin expression (Ferrucci et al., 2018; Bibbo et al., 2021). Furthermore, gene expression and gene ontology analyses identified other neurogenesis-related genes (i.e., OTX2, CYFIP1, and GLI2) as correlated to Prune1 (Ferrucci et al., 2018; Bibbo et al., 2021).

GR4 MB comprises >40% of all MB and is stratified in three subtypes that show no differences in the overall survival or rate of metastatic dissemination at diagnosis. MYCN amplification is highly enriched in GR4- α tumors. GR4- α and GR4- γ show enrichment of chromosomes 8p loss, 7q gain and focal CDK6 amplifications. GR4- β MB are strongly enriched for SNCAIP duplication, almost ubiquitous i17q and GFI activation (Cavalli et al., 2017).

Furthermore, a subtype-specific enrichment of cytogenetic and focal copy number aberration derived from the DNA-methylation array data set further stratified GR3 and GR4 MB into eight distinct molecular subtypes (Sharma et al., 2019). More in details, subtype I was defined with a balanced genome with OTX2 amplification. Subtype II was characterized by enrichment of gain of chromosome (chr) 8, chr13q, chr1q and MYC locus. Subtype III was enriched for loss of chr8p and chr10q, while subtype IV by losses of chr8, chr10, chr11, and chr13. The subtype V exhibited i17q, chr16q loss and amplification of both MYC and MYCN. Subtypes VI and VII demonstrated gain of chr7 and loss of chr8. Subtype VIII exhibited a relatively balanced genome with i17q. MYC amplification was mostly found in subtype II and III (Sharma et al., 2019). Then, the addition of molecular risk markers including methylation and whole chromosomal aberration (WCA) have identified a new prognostic stratification among the eight subtypes (I-VIII) of “non-WNT/non-SHH” MB (Mynarek et al., 2022). The patients considered as “very low risk” group include those belonging to subgroup VII in a clinical standard risk background and account for 6% of patients with “non-WNT/non-SHH” MB. In contrast, patients with a clinical “high-risk” profile that belongs to subgroup II, III or V among the “non-WNT/non-SHH” MB constitute a group of “very high-risk” patients for relapse (Mynarek et al., 2022).

Of interest, distinct developmental niches for all four major subgroups were identified, thus linking each subgroup to a cell-specific ancestor.

Through human single cell RNA sequencing (scRNA-seq) of fetal cerebellar data, the interplay between oncogenic events and putative cells of origin for each MB subtype were mapped (Williamson et al., 2022). In this regard, MB occurs in various neuronal stem or progenitor cell populations according to the consensus subgroup. Mutations that activate WNT signaling lead to WNT MB in the lower rhombic lip progenitor cells (Gibson et al., 2010; Jessa et al., 2019). In contrast, mutations that activate Sonic hedgehog signaling leads to SHH MB in the upper rhombic lip granule cell lineage (Yang et al., 2008). Recently, the rhombic lip subventricular zone (RL^SVZ^) giving rise to unipolar brush cells, has been postulated to be the common cellular origin of GR3 and GR4 MB *via* multi-omics approach mapping of MB subgroups in the context of human fetal cerebellar development (Phoenix, 2022; Smith et al., 2022). Furthermore, these tumors are driven by the disrupted function of the core binding factor alpha (CBFA) complex that recruits epigenetic modifiers, with mutually exclusive variants in CBFA2T2, CBFA2T3, PRDM6, UTX and OTX2 loci in GR4 MB (Hendrikse et al., 2022; Phoenix, 2022).

Thus, the identification of different genetic MB subtypes with distinct clinical behavior and “risk profile” should allow for more precise and rational planning of clinical trials with a personalized approach in MB affected children.

### Epigenetics in MB: DNA methylation and histone modifications

MB tumorigenesis has been reported with predominant epigenetic alterations consisting of large hypomethylated chromosomal regions that cause increased gene expression (Pugh et al., 2012; Hovestadt et al., 2014). It is estimated that more than 30% of MB samples, depending on the subgroup, are mutated in those genes encoding for epigenetic regulators, thus suggesting that epigenetic alterations are a very important part of MB progression. Each MB subtype may require different sets of chromatin remodelers and histone modifiers driving different transcription programs (Yi and Wu, 2018). The epigenetic regulators that mostly affect MB are DNA methylation and histone modifications and in the following chapter we will discuss further.

### DNA methylation

DNA methylation has also been shown to play an essential role in various physiological and pathological processes. Hypomethylation of bulk genomic DNA and hypermethylation of CpG islands have been implicated in the initiation and progression of human cancer, including brain tumors. In this regard, hypermethylation may lead to transcriptional repression of tumor-suppressor genes. On the other hand, hypomethylation of promoter sequences may reactivate the expression of silenced oncogenes, thus linking epigenetic and genetic mutational statu, both of great impact for therapy. Tumor-specific methylation changes have been established as prognostic markers in many tumor entities. In fact, in a methylome study, conducted on n.230 MB patients, four DNA methylation subgroups related to their transcriptomic counterparts (WNT, SHH, GR3 and GR4) have been found (Schwalbe et al., 2013).

The repression of tumor suppressors, in the various MB subgroups, mostly occurs through the hypermethylation of the CpG islands along their promoters. Indeed, several tumor-suppressors genes, including RASSF1, CASP8, HIC1 and ZIC2, were shown to be frequently epigenetically inactivated by hypermethylation in their promoter regions in MB (Lusher et al., 2002; Rood et al., 2002; Zuzak et al., 2002; Pfister et al., 2007).

Furthermore, the methylation of MXI1 and IL8 loci have been identified as high-risk biomarkers in non-WNT MB patients, thus improving disease-risk stratification (Schwalbe et al., 2013). Also, a cross-species approach for the study of methylation status in n.216 sub-grouped human and n.4 murine MB genomes has identified VAV1 as an epigenetically regulated oncogene with a key role in tumor maintenance and associated with a poor outcome in the SHH subgroup (Lindsey et al., 2015). In details, the widespread regional CpG hypomethylation of VAV1, leads to its elevated expression, thus underlining its potential as a therapeutic target and prognostic biomarker in SHH-MB (Lindsey et al., 2015). Epigenetic mechanisms responsible for MB tumorigenesis were also found in the sonic hedgehog pathway. In this regard, one of the mechanisms responsible for the low expression levels of the negative regulator of the sonic hedgehog pathway (HHIP) is ascribed to hypermethylation mechanisms in MB (Shahi et al., 2010; Shahi et al., 2011).

Of interest, *via* integrated analysis of bisulfite sequencing, RNA-seq and ChIP-seq data obtained from n.34 human MB samples, different hypomethylated regions downstream of promoters (extending tens of kilobases in the gene body) were found responsible for increased gene expression, rather than gene silencing, across MB subgroups.

An additional example is related to the miRNA-processing gene LIN28B, resulting in alternative promoter usage and/or differential messenger RNA/microRNA expression. Hypomethylated in LIN28B locus is associated with higher mRNA expression in GR3 and GR4 MB. LIN28B is known to regulate multiple oncogenic processes by downregulating the tumor-suppressive LET-7 miRNAs whose expression is low in GR3 and GR4MB subgroups. LIN28B expression has been found correlated with poor prognosis in neuroblastoma (Chen et al., 2020) and this also held true for GR3 and 4 MBs (Hovestadt et al., 2014).

Furthermore, somatic mutations and number aberrations affecting the histone code-modifiers causing a global change in the chromatin state were also reported in GR3 and GR4 MB. In a study by Dubuc et al. were characterized several alterations that converge on modifiers of H3K27-methylation, including EZH2, KDM6A, KDM6B. Indeed, mutually exclusive mutations in MLL2 and its binders KDM6A, which are both involved in gene expression activation through H3K4me3 accumulation and H3K27me3 removal (Schuettengruber et al., 2007), respectively, were found in GR4 MB (Dubuc et al., 2013).

### Histone modifications

The complex network of histone modifications including acetylation, methylation, phosphorylation, and ubiquitination alters histone chromatin structure locally into the genome by recruiting protein effectors which are known to control the genetic transcription machinery. Numerous studies have found alteration of the genes that code for the enzymes responsible for the epigenetic modification of histone proteins in the various MB subgroups (Northcott et al., 2009).

In particular, Yi et al. identified homozygous deletions and recurrent focal amplifications in genes responsible for the methylation/demethylation of lysine at position 9 of histone 3 (H3K9): including L3MBTL3, L3MBTL2 and SCML2 (polycomb proteins), EHMT1 and SMYD4 (methyltransferase), and JMJD2B and JMJD2C (demethylases). Confirming this finding, the 40% of medulloblastoma samples show lower global H3K9me3 levels than normal (Yi and Wu, 2018).

Other important sites for chromatin regulation involve the methylation/demethylation of H3K4 and H3K27. In this regard, MLL2/KMT2D and MLL3/KMT2C complexes are required to maintain H3K4me1 levels in enhancers (Rao and Dou, 2015; Sze and Shilatifard, 2016), thereby modulating enhancer activities during development and in cancer. In 16% of MB with recurrences in SHH and GR 4 subgroups, inactivating mutations in MLL2/KMT2D and MLL3/KMT2C (two lysine methyltransferases that enhance H3K4me2/3) are found, thus suggesting these genes could act as tumor suppressors in MB (Roussel and Stripay, 2018).

The UTX/KDM6A protein is a specific demethylase of the repression marker H3K27me3 which interacts with the MLL2/3 complexes (specific H3K4) (Cho et al., 2007; Lee et al., 2007). In fact, the two subunits MLL2/3 and UTX can destabilize the epigenetic structure by methylating H3K4 and demethylating H3K27me3 mutually exclusive in MB (Dubuc et al., 2013). It is additionally known that 4% of MBs have homozygous mutations or deletions of UTX/KDM6A usually in male patients, but are also present in female patients in case they have lost an X chromosome (Xp11.3), with a greater enrichment in GR4 tumors (Dubuc et al., 2013; Jones et al., 2013).

PRC2, also known as “polycomb repressive complex 2,” plays an important role in cell differentiation, identity maintenance and proliferation (Boyer et al., 2006; Lee et al., 2006) and together with PRC1 is part of the polycomb protein complex (or PcG). This protein complex is a histone methyltransferase of H3K27 that has been found to be dysregulated in many tumors and can lead to oncogenic or tumor-suppressive activity depending on the cellular context. The PRC2 complex is composed of several subunits (EZH2, EED, SUZ12, JARID2 and Rbap46.48), which are often amplified in MB (Bunt et al., 2013; Dubuc et al., 2013), and their actions leads to an elevated level of H3K27me3 by repressing specific tumor suppressor genes, thus facilitating tumor development (Greer and Shi, 2012).

However, some cancers (e.g., myeloid leukemia) are associated with loss-of-function mutations of the PRC2 complex and associated to low expression levels of H3K27me3 (Hock, 2012), which drives the expression of specific oncogenes (Schwartzentruber et al., 2012; Wu et al., 2012). In this regard, several studies have reported that the GR3 and GR4 MB subgroups are characterized by a combination of histone markers consistent with a stem/progenitor cell-like identity with high levels of EZH2 expression, marked by H3K27me3 and impaired H3K4 methylation (Dubuc et al., 2013). The GR3 subset of MB can also be distinguished by acetylation at H3K27(ac) and methylation at H3K4me1 a marker of active enhancers (Northcott et al., 2009; Robinson et al., 2012). Of interest in MBs, EZH2 overexpression and complete loss of KDM6A/UTX are mutually exclusive, thus suggesting a primary role for H3K27me3 in MB (Dubuc et al., 2013). Thus, an exhaustive simplified model would suggest that EZH2 could act an oncogene, while KDM6A/UTX could bact as a tumor suppressor. Contrary to expectations, the deletion of EZH2 and SUZ12 in both mouse and human GR3 MB *via* TALEN and CRISPR-Cas9 gene editing is able to accelerate the tumor progression, suggesting a tumor suppressor activity of the protein complex in the tumor (Vo et al., 2017).

Another important component in the epigenetic regulation is the acetylation/deacetylation that occur at the histone level operated by acetyltransferase (HAT) and deacetylases (HDAC).

The transfer of acetyls into the amino-terminal tails of histones by HATs results in the relaxation of DNA-histone interactions promoting gene transcription. Although HATs play a crucial role in brain development, little is known about their role in MB and it remains an underexplored field. HATs play a crucial role in brain development, and downregulation of the H4K16 HAT, hMOF, has been associated with poorer outcomes in MB patients (Pfister et al., 2008).

The CREBBP and EP300 (HAT) genes encoding CBP and p300, respectively, are enzymes that catalyze acetylation in histone H3 of lysine 27 (H3K27), a marker of enhancer activation, are mutated in some MB patients. Thus, activating the expression of many genes involved in tumor development and progression, although at this time the molecular targets of CBP and p300 have not yet been found (Ong and Corces, 2011) (Jin et al., 2011; Jones et al., 2012; Pugh et al., 2012; Robinson et al., 2012). A significant overrepresentation of somatic alterations targeting HAT complexes was found in the SHH subgroup, as compared to other MB subgroups (Northcott et al., 2017). At this time, CREBBP, KAT6B and EP300 genes, as well as the regulatory components of the HAT complexes BRPF1 and KANSL1, show recurrent mutations, mostly limited to the SHH subgroup (almost 19% of patients with SHH MB) (Northcott et al., 2017). The mechanisms by which deregulation of HAT activity cooperates with constitutively active SHH signaling remains poorly defined, warranting further studies to determine whether this epigenetic pathway can be exploited therapeutically.

Furthermore, one important effector the active enhancer and super-enhancer (SE) landscape in MB was dissected by performing a ChIP-seq study for H3K27ac and BRD4 coupled with tissue-matched DNA methylation and transcriptome data across n.28 primary specimens (Lin et al., 2016). Indeed, enhancers regulating the receptor tyrosine kinase ALK were found highly active in the WNT MB; while enrichment of neuronal transcriptional regulators in GR4 and TGF-β signalling in GR3 (*via* focal amplification at the activin receptor type 2A “ACVR2A locus” which is a member of the TGF-β family protein receptors involved in binding and activating the SMAD transcriptional regulators) were identified *via* functional pathway analysis (Lin et al., 2016). The study of the SEs showed activation in ALK in WNT MB, SMO and NTRK3 in SHH subgroups, LMO1, LMO2, and MYC in GR3, and ETV4 and PAX5 in GR4 (Lin et al., 2016). Thus, the analysis of chromatin landscape in MB showed the molecular subgroups with differentially regulated enhancers and SEs, mostly inferring master transcriptional regulators as responsible for their specific divergences.

Altogether, these insights demonstrate the critical importance of epigenetic analyses of primary tumors to highlight the core regulatory circuitries, especially in poorly characterized and clinically heterogeneous GR3 and GR4 MB malignancies, thus envisioning the future use of epigenetic drugs against these “high risk” tumors.

### MB tumor microenvironment

The tumor microenvironment is considered a complex network implicated in the communication between tumorigenic cells and non-cancerous cell types, including endothelial cells, pericytes, fibroblasts, and immune cells (Spano and Zollo, 2012). The brain has been long considered an “immune privileged” organ sheltered from immune cells; however, the immune privilege concept has been recently revised for the brain (Quail and Joyce, 2017). In fact, in certain brain tumors, including MB, the blood brain barrier is often compromised and there can be a robust infiltration of immune cells from the peripheral circulation (Quail and Joyce, 2017). Thus, the brain tumor microenvironment contributes to tumorigenesis and metastatic spread also in MB (Quail and Joyce, 2017).

Regarding the BBB, it is also responsible for the different prognosis among the subgroups, due to the tumor vessel phenotype (Phoenix et al., 2016). The majority of the patients with WNT MB show a good prognosis, also due to their unique vascular phenotype caused by a large amount of WNT antagonists (e.g., WIF1 and DKK1) secreted by endothelial cells that block WNT signaling in a negative feedback loop (Northcott et al., 2011). This paracrine axis is responsible for the ‘leaky’ fenestrated vasculature of the blood–brain barrier (BBB) in WNT MB that allows high levels of intra-tumoral chemotherapy to accumulate, thus promoting a robust therapeutic response. In contrast, SHH MB, contains an intact blood brain barrier, thus rendering this tumor more resistant to chemotherapeutics and more aggressive (Phoenix et al., 2016).

There is a growing body of evidence that the immune system in the tumor microenvironment has both positive and negative effects on tumor development, as has also been reported for brain cancers (Quail and Joyce, 2017; Klemm et al., 2020). The immune cells identified in brain tumors are mainly macrophages (BMDMs, CD45high, CD49D/ITGA4+), tissue-resident microglia (CD45low, CD49D/ITGA4-, (Lisi et al., 2017; Quail and Joyce, 2017), T cells, B cells, NK cells (Masson et al., 2007; Geller and Miller, 2011), Myeloid-Derived Suppressor Cells (MDSCs (Abad et al., 2014); and Dendritic Cells (DCs; (Quail and Joyce, 2017).

In MB, the low percentage of “effector” T cells, such as granzyme B-expressing CD8^+^ T cells and natural killer (NK) cells have suggested a very low level of active antitumor immune responses in MB (Bockmayr et al., 2018; Vermeulen et al., 2018). Notwithstanding the limited number of infiltrating immune cells in MB, emerging evidences have identified mechanisms of immune evasion in BTME (Eisemann and Wechsler-Reya, 2022).

In this regard, the loss of MHC class I antigen exposure on tumor cell surface is a common mechanism of immune escape that has been yet reported (Vermeulen et al., 2018). Furthermore, recent studies have demonstrated minimal infiltration of activated NK cells (Vermeulen et al., 2018), mostly due to the downregulation of NKG2D ligands on tumor cells (Haberthur et al., 2016). This action is due through the release of the immunosuppressive cytokine transforming growth factor β (TGF-β) (Gate et al., 2014; Bockmayr et al., 2018; Powell et al., 2019). Of interest, TGF-β, mainly expressed in metastatic GR3 MB (Ferrucci et al., 2018) drives the conversion of conventional CD4^+^ T cells (T-conv) to immunosuppressive regulatory T cells (T-regs). These cells have been also correlated to resistance to immunotherapeutic approach with checkpoint inhibition in brain cancers (Amoozgar et al., 2021). T-regulatory cells (T-reg) infiltration has been described within the BTME (Gate et al., 2014; Bockmayr et al., 2018; Vermeulen et al., 2018 #83; Grabovska et al., 2020) and in those chemotherapeutic-treated patients from their peripheral blood analyses (Gururangan et al., 2017). A higher count ratio between neutrophil to lymphocyte has been also reported, thus reflecting systemic immunosuppression (Bockmayr et al., 2018). Furthermore, a crosstalk between astrocytes, microglia and myeloid cells has been also described. In this regard, SHH MB cells have been reported with the ability to transdifferentiate into interleukin-4-(IL-4) secreting astrocytes, which stimulate microglia to release insulin-like growth factor 1 (IGF1) together with the immunosuppressive IL-10 (Yao et al., 2020) both of great importance on mediating tumor progression.

Additionally, in GR3 MB, tumor-associated astrocytes produce high levels of CCL2 chemokine (Liu et al., 2020) that has been also shown to promote leptomeningeal metastasis (Garzia et al., 2018) and to act as a chemoattractant for other immune cells, including monocytes and bone marrow-derived macrophages (Maximov et al., 2019) with a tumor-promoting function, showing definitively an M2-like gene expression profile in SHH MB model (Margol et al., 2015). This phenomenon was observed additionally in GR4 MB (Grabovska et al., 2020) tumors. Thus, the tumor promoting function of MB-infiltrating macrophages was also confirmed in different mouse models of SHH MB (Atoh1-SmoM2, see (Tan et al., 2021); Ptch1+/−; Tp53−/−, see (Dang et al., 2021). The presence of immunosuppressive myeloid derived suppressor cells “MDSCs” has also been reported for SHH MB model where an increase infiltrating immunosuppressive regulatory T cells (T-regs) and a reduce effector T cells (T-conv) was identified (Abad et al., 2014).

As in the contest of immune checkpoints regulations, PD1 expressing T cells and PD-L1 positive cells are limited in number in MB GR3 but yet expressed in SHH MB models (Pham et al., 2016), thus leading to immune escape of tumor cells by promoting T-cell exhaustion (Dong et al., 2002).

Recently, through single-cell RNA sequencing (scRNA-seq) performed on twenty-eight primary childhood MBs, the spectrum of immune infiltrating cells in MB has been described. These results showed that only M2-myeloid cell proportions were different between subgroups, being more abundant in infant SHH than GR3 and GR4 MB patients. In contrast, none of the lymphocyte subpopulations (NK, B cells and regulatory T-regs) were significantly differentially distributed across MB subgroups in the scRNA-seq cohort (Riemondy et al., 2022). Although MB had been a long considered a “cold immune response tumor,” there are several evidences that this is not the case. Altogether, these pieces of evidences indicate that MB cells can be susceptible to immune-mediated attack (Eisemann and Wechsler-Reya, 2022).

Thus, the identification of infiltrating immune cells with immunosuppressive phenotypes in the BTME of MB patients opens the door for immunotherapeutic strategies for MB treatment in the near future.

### Targeting the immune cells in MB tumor microenvironment

Cancer immunotherapy is a burgeoning field for targeted therapies that can harness the cytotoxic potential of the immune system against tumorigenic cells. Several immunotherapy approaches for children with malignant brain tumors are underway now.

Pre-clinical investigations in MB have demonstrated that macrophages can be unlocked to phagocytose MB cells using a humanized monoclonal antibody that targets CD47 on the MB cell surface and impairs its interactions with the SIRPα receptor on myeloid cells, analyses performed in GR3 MB models (Gholamin et al., 2017). Other studies using “preclinical models of MB” (comparing GR3 MB and SHH MB tumors) have indicated that GR3 MB has higher levels of PD1+ CD8+ T cells, and correspondingly, a more pronounced response to PD1 blockade (Pham et al., 2016). In contrast, in an additional study (Vermeulen et al., 2018) including seventeen (n.17) MB tissues, there were limited numbers of PD1+ T cells and an absence of PD-L1 expression, which thus suggested limited value for immunotherapy with PD1/PD-L1 blockers (as checkpoint inhibitors).

Of interest, in a cohort of eighteen (n.18), additional MB patients the number of circulating T-regs increased during radiation and chemotherapy, with substantial reductions in the overall lymphocyte counts (Gururangan et al., 2017). Currently, the data are controversial related to the amount of T lymphocytes present in MB TME. Thus, the definition of “cold” tumor at this time should be strongly reconsidered.

Also of note, MB cell lines have been shown to express specific ligands that trigger NK-cell- activating receptors and are thus susceptible to NK-cell-mediated cytotoxicity (Castriconi et al., 2007). However, the high expression of HLA class I on tumor cell (ATSS: HTB-186) makes them resistant to NK-cell cytotoxicity. Indeed, blocking HLA class I on MB cells and/or IL-15– stimulated NK cells can overcome the inhibitory effects mediated by HLA class I overexpression on tumor cells (Fernandez et al., 2013).

The presence of MDSCs were also investigated in MB, using a murine model that develops spontaneous cerebellar tumors resembling pediatric MB (i.e., Smo mutant mice). MDSCs were present in premalignant lesions in these mice and were highly abundant in fully developed MB tumors. The recruitment and activation of these MDSCs was shown to be driven by the STAT3 activation pathway. Indeed, the deletion of STAT3 in myeloid cells increased the proinflammatory phenotype of peripheral macrophages and resulted in a strong reduction in the abundance of MDSCs and Tregs within tumors, which increased the relative proportion of T effector cells (Abad et al., 2014).

Of importance, promising data are coming from Phase I and II clinical trials currently underway (NCT01326104) where RNA-loaded DCs and activated T cells in patients with medulloblastoma.

Briefly, autologous dendritic cells (obtained from leukapheresis of MB patients) were maturated *in vitro* and loaded with a personalized cohort of total tumor mRNA (amplified from a personalized cDNA library) representing a tumor-specific transcriptome.

These RNA-loaded DCs were then cultured with autologous T cells (also obtained *via* leukapheresis), to activate them and re-administered to patients with MB (Nair et al., 2015) together with the activated T cells.

Recently, CCL2 (MCP-1) chemokine (also known as monocyte chemoattractant protein-1; MCP-1) was shown to induce MB leptomeningeal metastatic dissemination by acting in concert with its receptor CCR2, which is on macrophages and glial and endothelial cells (Garzia et al., 2018). In the brain TME, CD163+ (M2-polarised) macrophages/microglia were since reported to be the major source of CCL2, the widely recognized effects of which include recruitment of CCR4+ T-regs and CCR2+ MDSCs (Chang et al., 2016). Targeting CCL2 represents a novel therapeutic strategy for brain cancer immunotherapy. Among the CCL2 inhibitors, immunomodulatory drugs (IMiDs), which include pomalidomide (NCT03257631), affect various molecular and cellular elements within the TME, and also the levels of various tumor-supporting cytokines, including CCL2, thus modulating monocytes, T cells and NK cells (Chanan-Khan et al., 2013). Pomalidomide crosses BBB (CNS penetration, ∼39%; (Li et al., 2013), and preclinical evaluation has shown its impact on the brain TME, where it causes increased macrophages and NK cells, decreased M2-polarised TAMs, and increased M1-polarised TAMs with phagocytic activity (Li et al., 2013). Indeed, a phase 2 clinical study to investigate the efficacy of pomalidomide for children with recurrent or progressive primary brain tumors, including MB, has been performed (Fangusaro et al., 2021), NCT03257631).

At present, there are a growing number of new immunotherapeutic approaches under investigation including immune checkpoint inhibitors, oncolytic viruses, cancer vaccines, chimeric antigen receptor T cell therapies, and natural killer cells in recurrent and refractory MB patients.

In this regard, notwithstanding the limited PD1 expressing cells in MB environment, PD1 blockade has been shown to be a more effective therapeutic outcome in GR3 MB mostly due to the higher percentages of PD1+ CD8^+^ T cells infiltration in this tumor subgroup. Thus, the checkpoint inhibitors against PD1 (e.g., nivolumab, pembrolizumab, and durvalumab, are each under investigation in clinical trials for MB and other CNS tumors (NCT03173950, NCT02359565, and NCT02793466) (Gorsi et al., 2019; Audi et al., 2021).

Oncolytic viral therapy reduces tumor burden by stimulating the innate immune response. Viruses are invaded and propagated within tumor cells. The lysed tumor cells could expose the tumor antigens to the immune system and stimulate the immune response to eliminate tumors. The oncolytic viral therapy based on the exposure of tumor antigens to the immune cells has been investigated in MB *in vitro*. Polio/rhinovirus recombinant (PVSRIPO) was reported to reduce the cell proliferation of GR3 MB cells expressing CD155 receptors (Thompson et al., 2018). Similarly, decreased cell proliferation of MB has been shown upon anti cytomegalovirus CMV drug (ganciclovir) alone or in combination with the COX-2 inhibitor celecoxib. (Baryawno et al., 2011; Yang et al., 2014).

Adoptive NK cell therapy is based on the re-administrating of immune cells “educated” to target cancer cells. Several *in vitro* approaches considering NK cells have been investigated in MB. In this regard, the activation of receptors natural killer group 2 member D activator receptor (NKG2D) (Castriconi et al., 2007) and the downregulation of TGF-β receptors were found to enhance the cytotoxicity of NK cells (Yvon et al., 2017) on brain tumor cells, including MB.

Recently, approaches based on engineered chimeric antigen receptor (CAR) T cells therapy have been taken into account for MB therapeutic options. CAR-T cell therapy is of interest because of its efficiency to target a chosen antigen on tumorigenic cells, thus overcoming the problems of the reduced number of infiltrating T cells in the BTME (Louis et al., 2011). Because of the high expression of human epidermal growth factor receptor 2 (HER2) in MB, the efficacy of HER2-BBz-CAR T cells in mice MB models has been demonstrated in eliminating MB cells with no toxicities (Nellan et al., 2018). Currently, several clinical trials are underway in recruiting MB patients. HER2-specific and EGFR-specific CAR T therapies are under investigation (NCT03500991, (Vitanza et al., 2021); NCT03638167).

### Epigenetic mechanisms besides the immunosuppression of the brain tumor microenvironment

In recent years, significant advances have been made in our understanding of epigenetic mechanisms within TME (Marks et al., 2016; Sylvestre et al., 2020). Different studies showed how the epigenetic dysregulation of gene expression correlates with the altered phenotype of tumor-associated cells (fibroblasts, immune cells; (Liu et al., 2017). In particular, cancer-associated fibroblasts (CAF) gene expression is regulated by simultaneous action of epigenetic mechanisms including changes in DNA methylation, altered binding of histone modifying enzymes and their cofactors and alterations in histone markers (Vizoso et al., 2015). Interestingly, it has been shown that local DNA hypermethylation and global DNA hypomethylation can regulate the CAFs transcriptional activity, leading to the conversion of normal fibroblast into pro-invasive fibroblast in several cancers (brain and neck, lung and breast cancer, [(Albrengues et al., 2015). An increased expression of DNMT3b, together with local hypermethylation of SHP-1, mediated this conversion (Albrengues et al., 2015). In this context, the tumor cells secrete the proinflammatory cytokine leukemia inhibitory factor inducing P300 histone acetylation which in turn activates the JAK1/JAK3 signaling pathway leading to DNMT3b overexpression (Albrengues et al., 2015).

Besides CAFs, the TME also includes several epigenetically regulated pro-tumoral and antitumoral immune cell subsets (including T-regs, MDSCs, and TAMs) thus defining the immuno-suppressive microenvironment (Yang and Wang, 2021).

Immunosuppressive proprieties of T-regs are mediated in part by DNA demethylation of specific demethylated region. Specifically, the expression of transcription factor FOXP3, crucial for T-reg development and function, is strongly dependent on the T-reg demethylated region (Lal et al., 2009).

Additionally, MDSCs exhibit both local hypermethylation and a global DNA methylation profile, as previously described in CAFs and cancer cells. The increase in the expression of the *de novo* DNA methyltransferase DNMT3A in MDSCs may be connected to the loci-specific methylation (Rodriguez-Ubreva et al., 2017). These results presented are now under further investigations.

Another key component of the TME is represented by Tumor Associated Macrophages (TAMs) that can either inhibit or support tumor growth depending on their polarization to classically activated macrophages (M1s) or alternatively activated macrophages (M2s), respectively (Gordon and Martinez, 2010; Amedei et al., 2020; Ferrucci et al., 2021). Numerous studies have demonstrated that the epigenetic signaling regulatory factors as presented in TME are involved in modulating M2 polarization (Niu et al., 2022). In particular, it has been shown that the DNA methylation, achieved by the DNMT1 enzyme, plays a critical role in M1 activation by suppressing KLF4 gene, a member of the KLF family of zinc finger transcription factors (Cheng et al., 2014). In addition, histone methylation, performed by the H3K27 demethylase JMJD3, and histone acetylation have also been demonstrated to contribute to the M2 polarization (Satoh et al., 2010).

Brain tumors are characterized by a diverse immunological microenvironment that interacts with cancerous cells through a complex network (Perus and Walsh, 2019). TAMs, neutrophils, T and B lymphocytes, NK cells, dendritic cells, and microglia (specialist macrophage-like cells in the central nervous system, CNS) are the immune cells that can either promote or inhibit tumor formation (Yang and Wang, 2021). Particularly, resident microglia and TAM are the most prevalent immune cells in certain MB subtypes (Bockmayr et al., 2018). It has been demonstrated that microglia could adapt themselves to the microenvironment (Cheray and Joseph, 2018).

The microglia have been described as the brain’s macrophages. Depending on the phenotype they will acquire (e.g., neuroprotective, response to lipid, influence neuropathic pain), the microglia will develop different transcriptional factor activation signatures (Petralla et al., 2021).

An oversimplified and generally accepted view suggests that microglia cells in the tumor can polarize into two different phenotypes. They may acquire a pro-inflammatory phenotype, or so-called classical activation (M1 phenotype), eliminating microorganisms or tumor cells and secreting proinflammatory cytokines, with a most prominent action of interleukins IL-23, IL-12, IL-6, IL-1β and tumor necrosis factor-alpha (TNF-α). On the opposite, they may also acquire an anti-inflammatory phenotype with the alternative activation (M2 phenotype) and becoming neurotoxic. The M2 phenotype of the microglia cells is associated with low expression of MHC-II, IL-12, and IL-23 and production of anti-inflammatory cytokines acting like TGF-β and IL-10 (Chen et al., 2019).

The acquisition of a mature microglial-specific phenotype, as well as microglia activation states in health and disease and polarization, are modulated by epigenetic mechanisms such as histone post-translational modifications (i.e., methylation, acetylation, and phosphorylation), DNA methylation or gene expression regulation by non-coding RNAs (Cheray and Joseph, 2018). In mouse peritoneal macrophages (PMs), the CNS-derived IL-4 controls the acquisition of the M2 microglia-phenotype and triggers H4R3 methylation by regulating the expression of the proliferator-activated receptor PPAR-γ (Kittan et al., 2013). While in human macrophages, M2 polarization is associated with the histone H3K4methylation (H3K4me), induced upon M-CSF (colony-stimulating factor) and IL-4 stimulation (Kittan et al., 2013). Additionally in mouse, the IL-4-activated microglia upregulates JMJD3to promote H3K27 demethylation necessary for IRF4 and Arg1 overexpression (Tang et al., 2014).

However, the demethylase activity of JMJD3 seems to promote M2 microglia polarization and represses M1 microglia polarization (Das et al., 2017). In contrast, the H3K27 histone tri-methyltransferase activity of Enhancer of Zeste Homolog 2 gene (EZH2), the catalytic subunit of the Polycomb repressive complex 2 (PRC2), promotes M1 microglia polarization but represses M2 microglia polarization (Arifuzzaman et al., 2017).

**FIGURE 1:**
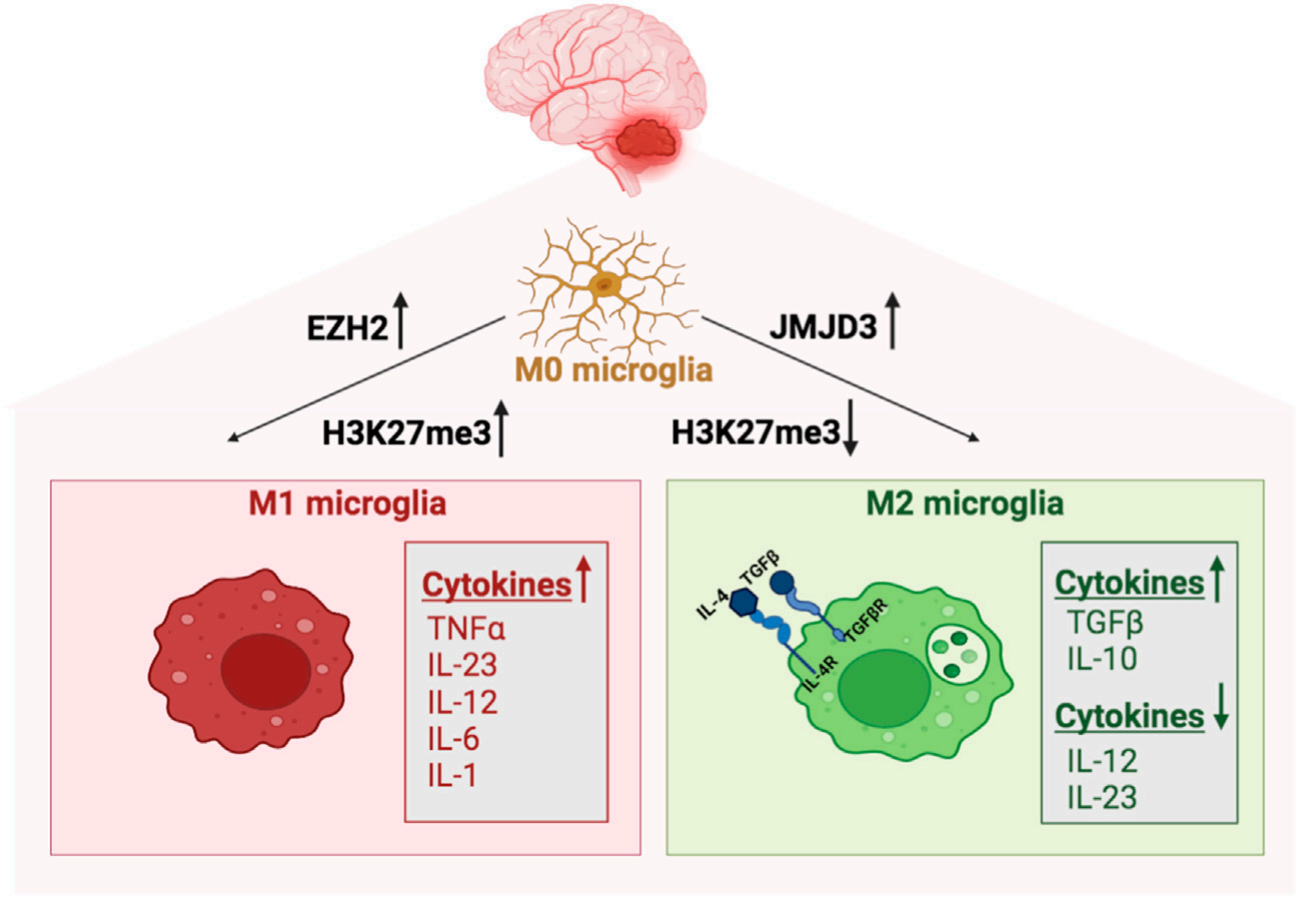
Epigenetic regulation of microglia in MB. Microglia polarize into M1 phenotype following the H3K27 histone tri-methyltransferase activity of EZH2. The M1 classical microglia secretes proinflammatory cytokines, with a most prominent action of interleukins IL-23, IL-12, IL-6, IL-1𝛃 and tumor necrosis factor-alpha (TNF-α). In contrast, the demethylase activity of JMJD3 promotes M2 microglia, which is associated with low expression of MHC-II, IL-12, and IL-23 and production of anti-inflammatory cytokines acting like TGF-𝛃 and IL-10.

Thus, epigenetic changes are associated with immunosuppressive modulation of those brain-resident cells (including microglia) in the BTME, as summarized in Figure 1.

However, some more specific factors, CSF-1 and IL-34, which are known to induce expression of microglial-specific genes (i.e., MAFB, MEF2C, SALL1 AND SPI1), are produced by neurons and astrocytes to allow the survival and renewal of microglia in brain tissues (Ponomarev et al., 2007). In turns, SPI1 (human) or Spi1 (murine) encode for PU.1, a microglial specific transcription factor; the neuronal-induced expression of PU.1 and other transcription factors like CEBPα, IRF8 and SALL1 leads to chromatin modification and generation of enhancers for gene expression, such as histone H3 K4monomethylated (H3K4me1) and histone H3 K9lysine acetylation (H3K9ac) (Cheray and Joseph, 2018).

Overall, a promising anti-tumor strategy against MB and brain tumors, could be the modulation and re-education of the set of immune-infiltrating cells in the BTME. Future studies will address these hypotheses.

### Epigenetic-based drugs for brain tumors: Histone methylation and acetylation modifiers

Clinical studies have shown that the development of pediatric brain tumors, including MB, can be significantly influenced by altered epigenetics (Maury and Hashizume, 2017). The ability to revert epigenetic modifications has proven useful in the development and improvement of therapies, resulting in the creation of a suitable treatment for these cancers. A new therapeutic approach has recently emerged targeting epigenetic modifiers. This approach includes inhibitors of both “writers” enzymes, such as DNA and histone methyltransferases, and of “erasers” enzymes, such as histone demethylase and histone deacetylases (HDACs). Also, the modulation of the “readers,” which are structurally varied proteins that identify and bind to covalent modifications of chromatin, is a novel method of targeting histone modification (Maury and Hashizume, 2017).

The Bromodomain and Extra-Terminal domain (BET) protein BRD4, which marks active enhancers and super-enhancers, represents an acetylation reader that has been extensively studied in brain tumors including MB (Wadhwa and Nicolaides, 2016). It has been demonstrated that BRD4 is pharmacologically inhibited by BET Inhibitor (BETi)-JQ-1, which in turn results in decreased proliferation and tumor growth in Sonic hedgehog (SHH) MB (Tang et al., 2014).

The JQ-1 has been described active in a human GR3 MB xenograft model *via* MYC downregulation resulting in a reduced tumor volume and a prolonged survival rate (Henssen et al., 2013). Nevertheless, JQ-1’s poor pharmacokinetic and pharmacodynamic characteristics prevent it from being used in clinical trials to treat MB at this time.

Currently, OTX015/MK-8628/Birabresib is a well-characterized CNS penetrant BET inhibitor (OTX015 (MK-8628) displays *in vitro* and *in vivo* antitumor effects alone and in combination with conventional therapies in glioblastoma models (Berenguer-Daize et al., 2016). Patients with recurrent glioblastoma were included in a Phase IIa trial as a result (NCT02296476). Interestingly, OTX105 has never been tested in MB yet and could be of an important value.

However, BETi, offers a potential way to monitor the advancement of cutting-edge MB therapy.

### Histone methylation modifiers

In addition to the epigenetic modifications due to readers in MB, reprogramming of DNA methylation patterns in these tumors using small molecule inhibitors of epigenetic enzymes, including EZH2 (Enhancer of Zeste Homolog 2), LSD1 (Lysine-specific demethylase 1) and DNMTs (DNA methyltransferases), could revert global hypomethylation of chromatin and increase expression of tumor suppressor genes (Zwergel et al., 2018).

The histone lysine N-methyltransferase EZH2 is a component of the Polycomb Repressive Complex 2 (PRC2), has been extensively researched in several cancers, including Glioblastoma and MB. In one of the earliest studies on MB, the relatively harmful 3-Deazaneplanocin (DZNep), an inhibitor of S-adenosyl-L-homocysteine hydrolase, was utilized as an unreliable and indirect EZH2i (Alimova et al., 2012). Also, Suva et al. pharmacologically inhibited EZH2 using the same compound which in turn led to a reduction in MYC expression and tumorigenicity in GBM (Suva et al., 2009). Moreover, Zwergel and collaborators have utilized MC3629 as a simplified homolog of two distinct SAM-competitive EZH2i EPZ005687 and GSK2816126 (Zwergel et al., 2018). This specific substance significantly impaired H3K27me3 and PCNA protein levels leading to apoptosis in human SHH MB cancer cell models but it also was effective in a SHH MB murine model (Zwergel et al., 2018). Importantly MC3629 has more effectively crossed the blood brain barrier both *in vitro* and *in vivo* and decreased H3K27me3 levels in the brain and cerebellum of mice with MB xenografts, thus resulting in a smaller tumor size and slowed tumor growth (Miele et al., 2017; Zwergel et al., 2018).

As mentioned above, histone/protein lysine demethylases (KDMs), which take away the methyl group (s) from a methylated lysine side chain, also dynamically regulates histone methylation. Currently, numerous irreversible KDM1A (LSD1) inhibitors have been identified and among them TCP, ORY-1001 and SP2509 are now evaluated in clinical trials as potential cancer treatments (Fang et al., 2019; Liang et al., 2020). Recently, LSD1 has shown to have a crucial role in the GFI1-mediated transformation of MB by binding to GFI1. Pharmacological inhibition of LSD1 with ORY-1001 successfully inhibited the growth of GFI1-driven tumors, suggesting therapeutic potentials of LSD1 inhibitors in GFI1-driven MB (Lee et al., 2019).

Moreover, it has been demonstrated that SP2509 hindered LSD1’s enzymatic activity rather than disabling the CoREST-LSD1 complex’s protein-protein interactions. By directly inhibiting LSD1, SP2509 was able to stop the growth of several human MB cell lines (DAOY, D283med, and ONS-76) (Inui et al., 2017).

Furthermore, an epigenetic modulator in cancer is represented by the DNA methyltransferases (DNMTs). In the last decades, DNA methyltransferase inhibitors (DNMTi) have emerged to target cancer-specific epigenetic aberrations. DNMTis are classified into two different groups: nucleoside analogs, that act as a natural substrate for DNMT (e.g., 5-azacytidine), binds to DNA and promotes the degradation of DNMT, and the Non-nucleoside analogs, which inhibit DNA methyltransferase activity through processes other than DNA incorporation (Hu et al., 2021).

Currently, Aza-20-deoxycytidine 5 phosphate has been tested in different phase I trials against various brain cancers, particularly in recurrent brain tumors, GBM, and ependymoma (Abballe and Miele, 2021). Recently, the effectiveness of zebularine, another DNMTi, was tested in four pediatric SHH-MB cell lines (DAOY, ONS-76, UW402, and UW473) (Andrade et al., 2017). Zebularine inhibited the development of MB cells by targeting the transcriptionally regulated GLI1, SMO, and PTCH1 members of the Sonic Hedgehog pathway (Andrade et al., 2017).

### Histone acetylation modifiers

One of the main targets for anticancer therapy among the different epigenetic modulators are the histone deacetylases (HDAC). The HDAC inhibitors appear to be the most promising and with the greatest prospect of success compared with previously described inhibitors. Initially, the first HDACi evaluated were broad-spectrum inhibitors, pan-inhibitors such as trichostatin A (TSA), valproic acid (VPA), hydroxamic acid suberoyl anilide (SAHA, vorinostat), capable of deacetylating multiple types of HDACs, in particular HDACs of class I and/or IIa/(Li and Seto, 2016; Hassell, 2019). Currently, most of the research has shifted toward screening for highly selective isoform-specific modulators, specific HDAC inhibitor (Zwergel et al., 2015). Anticancer effects of HDACi are usually non-specific pointing to aberrant alterations of long stretches of chromatin. The use of HDACi results in an extensive deacetylation which causes a transition from euchromatin to heterochromatin, thus affecting the cell proliferation, viability and differentiation, as well as migration and angiogenesis (Sanaei and Kavoosi, 2019).

Although to date, very few HDACi have been approved by the FDA, such as for drugs developed for rare T-cell lymphomas treatment (i.e., Vorinostat, romidepsin and belinostat), no HDACi has managed to enter clinical trials yet in MB therapy (Yi and Wu, 2018).

In the first studies, HDACi were tested on MB cell lines. MS-275 (Entinostat), a class I HDAC inhibitor, inhibited the proliferation of DAOY and D283-Med MB cell lines as well as of SHH and GR3- GR4 MB cell lines (Jaboin et al., 2002). Valproic acid (VPA), a known pan-inhibitor (HDAC class I and IIa/b), inoculated into the same cell lines, induced cell growth inhibition, cell cycle arrest, apoptosis, differentiation, and eventually (Li et al., 2005), while its systemic injection (400 mg/kg/day) in immunodeficient mice significantly inhibited the growth of xenografts of the same cell lines after 28 days of therapy. Such xenografts growth inhibition has result associated with the hyper-acetylation of histones H3 and H4 which in turn caused the suppression of TP53, CDK4 and c-MYC on the one hand, while on the other hand the activation of p21 (Li et al., 2005). Moreover, in MB primary cells induced by the synergistic combination of VPA and interferon-gamma (IFN-γ), caspase-8 expression is restored which in turn regulates TRAIL-mediated cell death (Hacker et al., 2009).

Furthermore, pan-inhibitors of HDAC such as SAHA, NaB and TSA have been shown to induce apoptosis related to permeabilization and subsequent dissipation of mitochondrial membrane potential and caspase-9 and -3 activation in DAOY and UW228 MB cells (i.e., SHH MB cellular model) (Sonnemann et al., 2006).

The action of HDACi (especially pan-inhibitors) also contributes to the enhancement of the cytotoxic effects of ionizing radiation in both primary and stabilized MB cells (Kumar et al., 2007).

SAHA has also been used in combination with chemotherapy used in the treatment of small cell lung cancer, etoposide/vincristine (IV), with different results depending on whether SAHA was given in combination with etoposide or vincristine. While in the first combination SAHA enhances the cytotoxic action; but not in the second (Sonnemann et al., 2006).

Combined treatment with HDACis toxin Helminthosporium carbonum (HC), SAHA and panobinostat of HD-MB03 cells (isolated from tumor material of a patient with metastatic GR3 MB) revealed high sensitivity to these HDACi, as well as a radiation sensitization with a significant increase in cell death following the concomitant treatment therapy (Milde et al., 2012).

The DKK1-encoded protein Dickkopf-1, a Wnt antagonist, is an epigenetically silenced tumor suppressor gene in MB. TSA treatment restores Dickkopf-1 expression in MB D283 Med cells (Vibhakar et al., 2007). Furthermore, TSA, in MB ONS-76 cells, has been shown to inhibit the activity of telomerase, an enzyme highly expressed in several tumors, regulate the cell cycle, upregulate the expression of p3 and p21, and reduce the levels of cyclin-D. Finally, in TSA-treated cells, pro-apoptotic effects are due to the upregulation of Bax and cytochrome C (Khaw et al., 2007).

Hedgehog (Hh)-induced upregulation of HDAC1 promotes deacetylation of GLI, an oncogene, and its transcriptional activation; the use of HDACi hinders the Hh-dependent growth of both neural progenitor cells and MB cells (Canettieri et al., 2010; De Smaele et al., 2011). The chimeric compound, NL-103, a dual inhibitor (HDACi and SHHi), exhibits a hybrid structure combining those of vismodegib, an FDA-approved smoothened receptor (SMO) inhibitor for other solid tumors, and SAHA, which is known to target the SHH signaling pathway by affecting the acetylation status of GLI1 and GLI2 (Canettieri et al., 2010). This novel, dual-target compound is able to inhibit the SHH signaling pathway by acting on two different targets, where it is more effective than treatment with single-target compounds (Zhao et al., 2014). Another important molecular target for regulating the Hh pathway is the selective inhibition of HDAC6 using three of its specific antagonists (Tubacin, CAY-10603 and ACY-1215). HDAC6 inhibition shortens the survival of induced MB cell lines and limits tumor growth in an *in vivo* allograft model (Dhanyamraju et al., 2015).

High-throughput screening of thousands of small molecules on the SHH-dependent murine MB cell line, SMB21, was undertaken to search for compounds to selectively inhibit class I HDACs as anticancer agents for SHH MB. Screening identified class I HDACi molecule, JNJ-26481585 (quisinostat) as a cell growth inhibitor of MB SHH tumors both *in vivo* and *in vitro* (Pak et al., 2019), while another study identified two class I HDACi, JNJ-26481585 and dacinostat that cause G2/M phase cell cycle progression blockade, cytotoxicity, and apoptosis. Furthermore, dacinostat and quisinostat attenuated xenograft MB growth in mice *in vivo* (Zhang et al., 2019). Cantieri et al., provided another explanatory example of the involvement of HDACi in the SHH signaling pathway by demonstrating that the HDAC1/2 selective inhibitors HDiA and HDiB blocked GLI1 and GLI2 activity through their acetylation and SHH MB cell growth in different SHH cell lines (Canettieri et al., 2010).

The administration of HDACi to tumor cells reactivates a whole series of gene pathways capable of sensitizing cells to apoptosis induced by chemotherapeutic agents (Hacker et al., 2011).

Overall, the several numbers of epi-drugs used in Phase I/II clinical trials or in preclinical studies (Table 1) demonstrate the value of utilizing these epigenetic inhibitors alone or in combination therapy for brain tumors.

**TABLE 1:** Classification of epigenetic drugs in preclinical or clinical trials for brain cancer.

Inhibitor	Target	Drug	Cancer type	Clinical trial identifier for brain tumors	References
BETi	BRD4	JQ1	SHH MB	—	Tang et al. (2014)
	Pan-BET	OTX015 (Birabresib)	Glioblastoma	NCT02296476 (Phase II)	—
EZH2i	EZH2	DZNep (3-Deazaneplanocin)	MB	—	Alimova et al. (2012)
			Glioblastoma		Suva et al. (2009)
		MC3629	SHH MB	—	Zwergel et al. (2018)
		EPZ-6438 (Tazemetostat)	MB	NCT03155620 (Phase II)	—
			Glioma	NCT03213665 (Phase II)	
KDMi	LSD1	SP2509	MB	—	Fang et al. (2019); Inui et al. (2017)
		ORY-1001			Lee et al. (2019)
					Liang et al. (2020)
DNMTi	DNMT1	5-aza-CdR (5-aza-2'-deoxycytidine)	Glioblastoma Ependymoma	—	Abballe and Miele (2021)
		Zebularine	SHH MB	—	Andrade et al. (2017)
HDACi	Pan-HDAC	SAHA (Vorinostat)	Glioma	NCT00994500 (Phase I)	—
			Recurrent MB	NCT01236560 (Phase II/III)	
	HDACs 1–4 HDACs 6–9	LBH589 (Panobinostat)	Glioma	NCT02899715 (Phase I)	—
			DIPG	NCT03566199 (Phase I/II)	
				NCT05009992 (Phase II)	
				NCT03632317 (Phase II)	
				NCT00848523 (Phase II)	
				NCT00859222 (Phase I/II)	
	HDACs 1–10	Belinostat	Glioblastoma	NCT02137759 (Phase II)	—
	HDACs 1, 2, 4, 6	Romidepsin	High-grade Gliomas	NCT00085540 (Phase I/II)	—
	HDACs 1–7 HDACs 9–10	VPA	DIPG	NCT00879437 (Phase II)	—
	HDACs 1, 3	MS-275 (Entinostat)	MB	—	Jaboin et al. (2002)
	HDACs	NL-103	SHH MB	—	Zhao et al. (2014)
	Pan-HDAC	JNJ-26481585 (Quisinostat)			Pak et al. (2019)

The type of inhibitor, the target, the name of the drug, the type of brain cancer, the phase of the clinical trial and the references or code of the clinical trial (https://clinicaltrials.gov) are listed. HDAC, histone deacetylase; DIPG, diffuse intrinsic pontine glioma; VPA, valproic acid.

The molecular subgroup stratification, together with the histopathological assessment, has been recently integrated into clinics to improve the therapeutic management of subgroup-driven MB.

## Discussion

The genetic and epigenetic heterogeneity of the MB microenvironment entangles the therapeutic development. To date, the current therapeutic strategies against MB still consist of maximal surgical resection followed by radio- and adjuvant chemotherapy with long-term side effects (Ezzat et al., 2016; Northcott et al., 2017; Northcott et al., 2019).

The molecular subgroup stratification has been recently integrated into clinics to improve the therapeutic management of subgroup-driven MB (Dubuc et al., 2013; Jones et al., 2013; Cavalli et al., 2017), including escalations doses of CSI, SHH inhibitors (Ezzat et al., 2016; Northcott et al., 2017; Northcott et al., 2019), the addition of carboplatin or intraventricular methotrexate, multi-agent chemotherapeutics (Gajjar et al., 2006; Packer, 2007; Jakacki et al., 2012; von Bueren et al., 2016). Efforts in reducing the dose or modifying the delivery of radiations (e.g., photon and proton beam radiation) are ongoing clinical trials (Thompson et al., 2020; Khan et al., 2021). However, the patients affected by GR3-GR4 MB are often already metastatic at diagnosis with a high risk of recurrence and very poor prognosis (Ezzat et al., 2016; Northcott et al., 2017; Northcott et al., 2019). Thus, improving therapeutic strategies less toxic and targeted to different risk groups, especially for those GR3-4 MB patients, still represent an unmet medical need.

To date, the risk stratification for MB remains a challenge although recent advances in the molecular understanding of the MB spectrum, *via* methylation and WCA, have provided novel risk markers, thus improving the molecular risk stratification among MB patients (Mynarek et al., 2022). Thus, the newly identified “low-risk” and “very high-risk” strata, accounting for 6%, and 21% of “non-WNT/non-SHH” MB patients, respectively, may improve future specific treatment. However, several unmet needs exist including which patients are more likely to respond to targeted therapies in MB and how novel immunotherapeutic approaches can mitigate the immunosuppressive nature of MB.

In this regard, the concept of CNS immune privilege has almost become obsolete, the brain environment continues to offer unique and formidable challenges to immune-based therapies.

The TME consists of a wide variety of cell types and extracellular components that make up an immunosuppressive environment that positively influences the development, progression, and relapse of tumors, including brain cancers (Quail and Joyce, 2017). MB has been a long considered a ‘cold’ tumor, due to the limited number of infiltrating lymphocytes (TILs) (Grabovska et al., 2020) (Bockmayr et al., 2018; Vermeulen et al., 2018). The most abundant immune cells in MB tumors are tissue-resident microglia and TAMs (Bockmayr et al., 2018), as also confirmed by scRNA-seq performed on twenty-eight primary childhood MB (Riemondy et al., 2022). Altogether, these lines of evidence indicate that MB cells can be susceptible to immune-mediated attack (Eisemann and Wechsler-Reya, 2022). However, immune evasion mechanisms have been reported for MB immunosuppressive environment (Eisemann and Wechsler-Reya, 2022) (Bockmayr et al., 2018) (van Bree and Wilhelm, 2022), including the loss of MHC class I on the tumor cell (Vermeulen et al., 2018), and the secretion of TGF-β (Gate et al., 2014; Bockmayr et al., 2018; Powell et al., 2019), especially in metastatic GR3 MB (Ferrucci et al., 2018) that acts as a chemoattractant for T-regs (Gate et al., 2014; Bockmayr et al., 2018; Vermeulen et al., 2018 #83; Grabovska et al., 2020) (Gururangan et al., 2017) and induces downregulation of NKG2D ligands on tumor cells (Haberthur et al., 2016), thus reducing the recruitment of activated NK cells (Vermeulen et al., 2018).

Furthermore, secretion of CCL2 (mainly by tumor-associated astrocytes (Liu et al., 2020)) in the BTME of MB was reported as responsible for leptomeningeal metastasis (Garzia et al., 2018), thus also promoting the recruitment of other immune cells, including those derived from the bone marrow-derived macrophages (Maximov et al., 2019). Thus, CCL2 represents a new target for MB treatment. In this regard, the anti-tumorigenic actions of an anti-CCL2 compound (i.e., Bindarit) were reported in other tumor types, to prevent the infiltration of M2-tumor-associated macrophages and MDSCs into the tumor microenvironment (Zollo et al., 2012).

Of interest, TGF-β has been also reported as enriched in metastatic GR3 MB (Lin et al., 2016). The role of TGF-β as an immunosuppressive cytokine in the TME has been already described (Gate et al., 2014; Bockmayr et al., 2018; Powell et al., 2019). Thus, pharmacological approaches targeting the genes/pathways related to the TGF-β signaling activation (e.g., including Prune1 or LSD1 inhibitors (Ferrucci et al., 2018; Zollo et al., 2022; Lee et al., 2019), should be investigated to inhibit the immunosuppressive BTME in MB.

To date, the characterization of the immune BTME in the MB subgroups remains unclear. Of interest, studies performed on murine models and human patients are showing some differences in infiltrating immune cells among the different subgroups, thus showing that murine SHH MB contained more dendritic cells, MDSC, TAMs, and TILs, whereas GR3 MB tumrs were composed of more CD8+ T-cells (Pham et al., 2016). Furthermore, analyses of the subgroup-specific immune microenvironment in human MB based on gene expression and cytokine secretion profiling confirmed the data from the animal models. In detail, human SHH-driven MB recruited more TAMs and T-cells, whereas GR3 and GR4 MBs contained more CD8+ T-cells and cytotoxic lymphocytes (Diao et al., 2020).

Of interest, numerous studies have shown that the various types of MBs have modifications in epigenetic regulator genes (Dubuc et al., 2013; Jones et al., 2013; Cavalli et al., 2017), which suggests that epigenetic alterations are a very important part for the appearance and progression of MB. *How epigenetic could help targeted immunotherapy in MBs?* The genetic and epigenetic heterogeneity of the cellular component of the MB microenvironment entangles therapeutic development. In-depth single-cell level profiling of biopsies may yield such insights defining access to therapy for subgroups of patients who may derive benefit from them highlighting the possibility of combinatorial treatment approaches targeting immunosuppression.

In recent years numerous epigenetic modulators, both of natural or synthetic origin, have been tested for the treatment of numerous types of cancer, but only some of them have been approved by the FDA and marketed, mainly for the treatment of hematologic malignancies (Mazzone et al., 2017) and a large body of *in vitro* experimental evidence has demonstrated that epigenetic drugs have broad cancer immunomodulatory properties.

These “epigenetic therapies” are based on the restoration of normal epigenetic signals by inducing cell growth inhibition, cell cycle arrest, apoptosis, differentiation and finally senescence without altering its genetic code (Li et al., 2005). Regarding MB, several epigenetic drugs are being tested in preclinical and clinical trials, especially for SHH MB (as listed in Table 1). A further step forward should be achieved by combining epigenetics with the chemotherapeutics currently used in clinics for MB management in lowering the dose and their related side effects of the chemotherapeutic regimens. Furthermore, future efforts should be done to test epigenetics drugs in the different molecular-driven MB subgroups based on the novel risk stratification.

**FIGURE 2:**
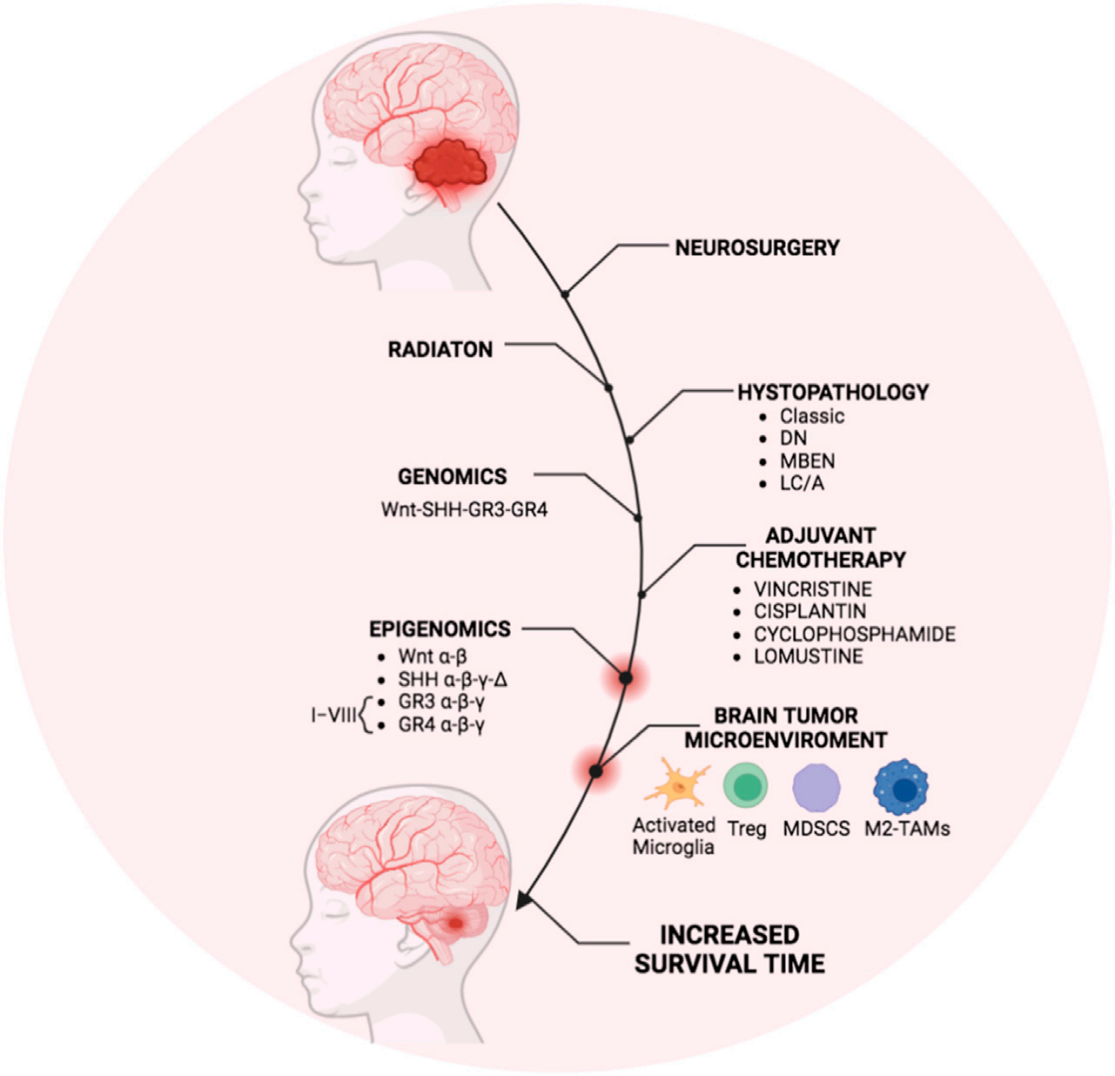
Overview of MB treatment modalities in the last decade. The scheme represents the therapeutic strategy for MB currently used in clinics that generally includes the surgical resection followed by irradiation and cycles of adjuvant chemotherapy mostly based on molecular and histopathological features. Recently, genomics and epigenomics approaches have clarified the molecular risk assessment among the molecular MB subgroups. Furthermore, the presence of immunosuppressive immune cells infiltrating the BTME in MB patients has been also reported. Thus, genetic and epigenetic modulators targeting the immune cells within the BTME could obtain the most efficient and personalized therapy to increase the patient survival timing. The figure was created *via* Biorender.com.

In conclusion, as summarized in Figure 2, in the last decade, the interplay between genetics and epigenetics has improved the molecular stratification of MB patients and the group risk assignment, thus defining new “risk” strata among the GR3- GR4 MB patients. These new molecular stratifications have ameliorated specific treatments.

Furthermore, the BTME is now emerging with immunosuppressive features and immune evasion mechanisms also in the “cold” MB environment, thus showing the presence of M2-TAMs and T-regs even if in a limited number. These findings are opening the door to the use of immunotherapeutic drugs or immunomodulator molecules also against MB.

Since epigenetic events have been described as of significance in modulating the phenotype of the immune cells in the BTME, the combination of epigenetics with immunotherapeutic drugs should be envisioned at this time to improve the survival timing of MB patients, especially in those belonging to the “very high risk” subgroup.

## Abbreviations

BBB, blood brain barrier; BTME, Brain tumor microenvironment; DN, desmoplastic/nodular; GR3, Group 3; GR4, Group 4; (LC/A), Large cell and anaplastic; MB, Medulloblastoma; MBEN, MB with extensive nodularity; OS, overall survival; SHH, Sonic hedgehog; TME, Tumor microenvironment; TAMs, Tumor-associated macrophages.
